# Revealing the significance of early detection of chronic obstructive pulmonary disease: insights from healthy lung initiative data at COP27 conference

**DOI:** 10.1186/s12913-024-11107-8

**Published:** 2024-07-31

**Authors:** Wagdy Amin, Rasha Ashmawy, Sandy Sharaf, Sally Zeina, Esraa Abdellatif Hammouda, Nancy Mohamed, Rewan Gamal, Dina Abu Hamr, Heba Gebril, Ola Alaa, Ussama Taha, Hazem El-Feel

**Affiliations:** 1 Director of the General Administration of Chest Diseases, MoHP, Giza, Egypt; 2 Department of Clinical Research, Maamora Chest Hospital, MoHP, Alexandria, Egypt; 3 Department of Biomedical Informatics and Medical Statistics, Medical Research Institute, Alexandria University, Alexandria, Egypt; 4 Clinical Research Department, El-Raml pediatric Hospital, Ministry of Health and Population, Alexandria, Egypt; 5 Department of Clinical Research, Kom-Elshokafa Chest Hospital, MoHP, Alexandria, Egypt; 6 Tanta Chest Hospital, MoHP, Gharbyia, Egypt; 7 Health Affairs, MoHP, Kafr Elsheikh, Egypt; 8 Chest Diseases Department, Dekernis Chest Hospital, MoHP, Dakahlia, Egypt; 9 Chest Diseases Department, Imbaba Chest Hospital, MoHP, Giza, Egypt; 10 General Administration of Curative Medicine, MoHP, Giza, Egypt

**Keywords:** COPD, Climate change, Smoking, COP 27, Pulmonary function test, PFTs

## Abstract

**Background:**

Climate change poses a significant threat to respiratory health, exacerbating conditions like asthma, Chronic Obstructive Pulmonary Disease (COPD), and respiratory infections increasing morbidity and mortality indirectly through exposure to greenhouse gases. During the 27th Climate Change Conference (COP27), the Healthy Lung Initiative (HLI) for the early detection of COPD among smokers was launched in Egypt.

**Objective:**

We aimed to detect the prevalence and predictors of confirmed COPD among smokers and assess physicians’ adherence to prescribing pulmonary function tests (PFTs) among the COP27 conference attendees.

**Methods:**

This study utilized smokers’ data from the HLI, employing a cross-sectional design with an interview-based questionnaire, supplemented by spirometry for lung function evaluation. Participants, comprising Egyptian and non-Egyptian residents in Sharm El-Sheikh city, were provided with educational materials and encouraged to seek further evaluation from a pulmonologist.

**Results:**

The HLI study, conducted from November 6th to 20th, 2022, involved 1133 participants, 90% (1047) of whom were smokers. Most smokers were males (96.3%) and Egyptians (98.6%), with half aged 30–39 and the majority starting smoking within the last 20 years. Despite common respiratory symptoms, 47% suffered from dyspnea on exertion, and only 4.3% have undergone lung function tests, suggesting a potential underdiagnosis of COPD. Most participants (82.6%) had an FEV1/FVC ratio > 70%, indicating no spirometry-confirmed COPD diagnosis, while 147 participants (17% of them) exhibited all three cardinal COPD symptoms simultaneously. Male gender and daily cigarette consumption were significant predictors for confirmed COPD, while age showed no significance in regression analyses.

**Conclusion:**

The ongoing HLI focuses on early detection and education to combat smoking-related risks, particularly among middle-aged males, while also highlighting the need for comprehensive strategies to address the intersection of smoking and climate change.

**Supplementary Information:**

The online version contains supplementary material available at 10.1186/s12913-024-11107-8.

## Introduction

Human health, particularly respiratory wellness, is in danger from climate change in several ways. It can directly trigger and worsen pre-existing respiratory diseases such as bronchial asthma, Chronic Obstructive Pulmonary Disease (COPD), rhinosinusitis, as well as respiratory tract infections (RTIs). Exposure to other risk factors, such as greenhouse gases, indirectly increases the morbidity and mortality of those individuals [1, 2]. Additionally, carbon dioxide (CO2) has a detrimental impact on air quality in a variety of ways, worsening heat waves, raising ozone levels, increasing air pollen and allergens, and causing forest fires, dust storms, and extreme weather events [3, 4]. Pollen season is extended, and air pollution is intensified due to global warming and elevated CO_2_ levels. More toxins, carcinogens, pollen grains, respiratory irritants, and pollutants are produced during forest fires. Ozone promotes bronchial hyperresponsiveness and worsens inflammation in the bronchial epithelial cells. All these variables worsen disease conditions, raise Emergency Department (ED) visits, hospitalizations, and lower lung function [4].

Egypt contributes only 0.57% of the world’s greenhouse gas emissions, but the country suffers greatly from climate change, a serious public health concern. This issue is recognized as one of the 17 Sustainable Development Goals (SDGs) worldwide due to its profound impact on people’s social and physical well-being. Although there is no particular emission reduction or limitation target that must be met, the national plan includes greenhouse gas reduction strategies [5]. Egypt hosted the 27th climate change conference (COP27) in November 2022, in Sharm El-Sheikh. During the conference period, the Egyptian Ministry of Health and Population (MoHP) launched the Healthy Lung Initiative (HLI) for the early detection of COPD among conference attendees especially smokers as a side health event [6].

There is a direct relationship between cigarette consumption and climate change. Pollution and climate change are considered environmental stressors and smoking itself is a coping mechanism for stress [7]. The harmful effects of smoking on one’s health have been extensively studied and recorded, but the effects of tobacco on the environment are often neglected. Every year, six trillion cigarettes are produced with consumption of nearly 5.8 trillion cigarettes annually [8]. Global smoking rates are rising as a result of increased smoking in developing countries. The steps of growing, curing, and manufacturing tobacco are harmful to the environment. More than 70% of the environmental harm caused by the entire process is attributable to farming, watering, and fertilizing. More carbon emissions are caused by burning coal and wood during the curing step than by all other stages combined [9]. In Egypt, where tobacco use is on the rise, the tobacco industry’s expansion is notable, with the country scoring 64/100 on the tobacco industry interference index in 2021, ranking 52/80 countries [10]. Rising smoking rates in Egypt, at 22% in 2010, driven by familial and peer influence along with exposure to Western media, underscore the urgent need for improved tobacco control measures to prevent serious health problems like cardiovascular diseases and COPD, requiring better healthcare worker training and standardized mass media and school-based health education programs [11].

COPD arises from a triad of genetic predisposition (G), environmental factors (E), and time (T), collectively known as GETomics, which can harm the lungs and disrupt their natural growth and aging processes [12]. Tobacco smoking, responsible for over 70% of COPD cases in high-income countries and 30–40% in low- and middle-income countries, along with household air pollution, poses a significant risk. COPD, a leading cause of long-term disability and mortality worldwide, is exacerbated by challenges in accessing healthcare, while the tobacco industry’s aggressive targeting of new customers in low- and middle-income countries is expected to escalate the global burden of COPD [13]. Symptoms experienced by COPD patients including dyspnea, chronic cough, activity limitations, sputum production, recurrent infections, and exacerbations are considered alongside relevant risk factors for diagnosis. According to GOLD guidelines 2024, COPD diagnosis is confirmed through spirometry, where a post-bronchodilator forced expiratory ratio (FER) of less than 0.7 indicates airflow obstruction in smoker-symptomatic patients. This ratio is calculated by dividing the Forced Expiratory Volume in one second (FEV1) by the Forced Vital Capacity (FVC) [14].

COPD is frequently misdiagnosed, leading to inadequate treatment for many patients, despite its preventability and treatability. To combat this, both community-based screening and thorough assessment of risk factors like age and smoking history are crucial for early detection [15]. At the United Nations COP27 conference, HLI employed these methods, collecting medical histories and offering counseling services alongside spirometry testing. This initiative highlighted the importance of addressing smoking-related health risks. The study aimed to determine COPD prevalence among conference attendees and evaluate physicians’ adherence to requesting Pulmonary Function Tests (PFTs) for early screening.

## Methodology

### Study design

This analysis utilized smokers’ data obtained from the HLI; a cross-sectional design using an interview-based questionnaire administered via a mobile application, with data collected by the interviewer. Additionally, spirometry is employed to evaluate lung function. Then participants were provided with educational materials and advised to seek further evaluation from a pulmonologist.

### Data source

The data were extracted from the HLI database, which aggregates information from public places including hotels, markets, and locations where people gather. The initiative was launched as a health-focused adjunct event during COP27, held in Sharm El-Sheikh, Egypt, spanning from 6th to 20th November 2022. This initiative featured a case-finding component aimed at facilitating the early detection of diminished lung function, which serves as a significant predictor for COPD among smokers. It collected demographic information including gender, nationality, and age, as well as smoking history details such as current smoking status, duration of smoking, and daily cigarette consumption. Additionally, it documented respiratory symptoms like exertional dyspnea, significant sputum production, chronic cough not related to colds, and whether participants had undergone pulmonary function testing in the past. Spirometry (SpirOx Plus®) was utilized to assess participants’ pulmonary function, estimating the FEV1/FVC ratio following bronchodilation, and these values were then incorporated into the participants’ data documented in the application-based questionnaire.

### Initiative study population

Egyptian and non-Egyptian residents in Sharm El-Sheikh city who were willing to participate in the study gave their verbal consent before being interviewed.

The following criteria were used to exclude individuals from participation in the study and undergoing PFTs:

1. Participants under the age of 16 and those who declined to take part in the study.
2. Individuals with acute respiratory or cardiovascular conditions.
3. Participants who had undergone recent surgery or experienced recent injuries.
4. Individuals with active respiratory infections.
5. Participants who had undergone recent eye surgery or experienced recent eye injuries.
6. Individuals with cognitive impairment or an inability to follow instructions.
7. Any other conditions that could potentially interfere with the accurate assessment of pulmonary function during the tests.

### Primary outcome

Evaluating the PFTs by measuring the FEV1/ FVC ratio after bronchodilation.

### Secondary outcome

Assessment of COPD prevalence and risk factors [COPD confirmed diagnosis by FEV1/ FVC less than 70% after bronchodilation among smokers [14]], as well as assessing the adherence of their chest physicians to ordering PFTs as a tool of diagnosis.

### Sample size and sampling

The sample size was calculated using epi info 7.2. software for population survey sample size calculation, using COPD prevalence 10.2% [16], population that attended the COP27 conference was 30.000, 2% margin of error, at 95% level of confidence the minimum accepted sample size was rounded to 870 participants.

### Statistical analysis

Descriptive statistics of the participant’s characteristics were presented as frequency and percentage, while PFTs data were represented as mean and standard deviation (SD). Chi-square (or Fischer exact test), and independent t-test were used for the comparison of the two groups.

Univariate and multivariate Logistic regression analyses were performed for the FEV1/FVC ratio including significant factors, identified in the bivariate analysis, to calculate adjusted and unadjusted odds ratio (OR) for each predictor. Data were analyzed using IBM SPSS [17], Version 25.

## Results

The total number of HLI participants was 1133, Supplementary Table S1. This analysis focused only on smokers, 1047 (90%). The majority of them were males and Egyptians (96.3%, and 98.6%, respectively). About half of the participants (47.9%) were aged 30–39 years old, more than two-thirds (72.8%) started smoking less than 20 years earlier, and 47.2% consumed 10–20 cigarettes per day. Regarding respiratory symptoms, dyspnea on exertion was mentioned by 47.5% of participants, 38.1% had sputum, and 26.8% had a cough without a cold. Only 4.3% were previously advised to perform lung function tests indicating the adherence of physicians to ordering PFTs for diagnosing COPD and/or its severity, Table 1.

**Table 1:** Description of the demographics and characteristics of the study participants (Smokers)

Variable (*N* = 1047)	Frequency	%	
Gender “Male”	1008	96.3%	
Nationality “Egyptian”	1032	98.6	
Age			
Less than 20 years	4	0.4	
20–29 years	146	13.9	
30–39 years	502	47.9	
40–49 years	267	25.5	
50–59 years	113	10.8	
≥ 60 years	15	1.4	
Smoking years			
Less than 20 years	762	72.8	
20–30 years	238	22.7	
More than 30 years	47	4.5	
Number of Cigarettes/ days			
Less than 10 cigarettes/day	255	24.4	
10–20 cigarettes/ day	494	47.2	
20–30 cigarettes/day	219	20.9	
More than 30 cigarettes/per day	79	7.5	
Dyspnea on exertion “Yes”	497	47.5	
Sputum “Yes”	399	38.1	
Cough without cold “Yes”	281	26.8	
Your doctor ordered a PFTs before “Yes”	45	4.3	

PFTs: Pulmonary Function Tests, FEV1: Forced Expiratory Volume at 1 s, FVC: Forced Vital Capacity

**Fig. 1:**
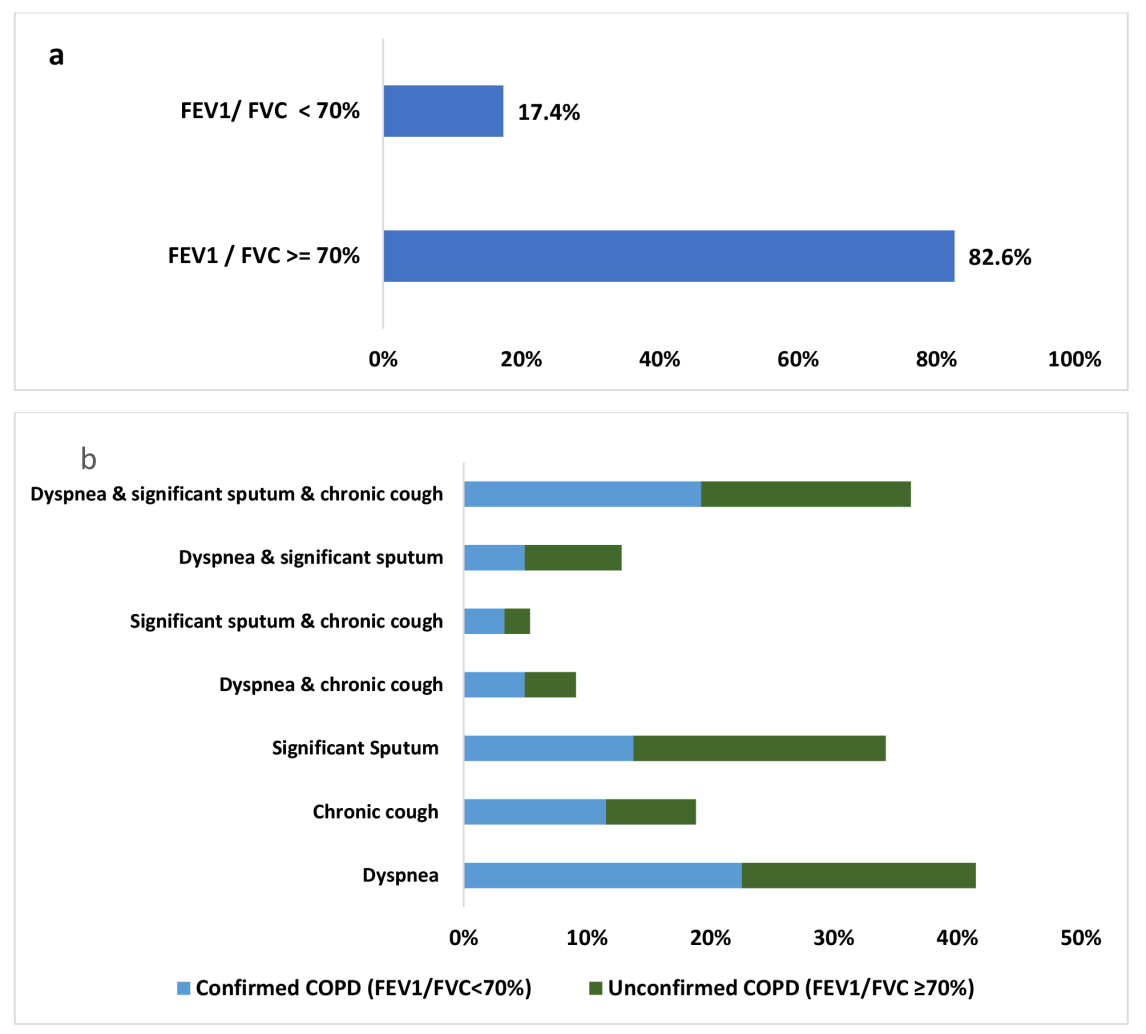
(**a**) Distribution of reduced FEV1/FVC ratio among smokers. (**b**) Distribution of COPD symptoms among smokers categorized by spirometry confirmation status

The mean FEV1%, FVC%, FEV1 /FVC% after bronchodilatation were 83.4%, 86.6%, 82.6%, Table 2. The majority of participants (82.6%) had FEV1/FVC ratio > 70%, Not spirometry confirmed COPD diagnosis, Fig. 1a. Approximately 17.4% of participants (182) exhibited the three cardinal symptoms of COPD together, whereas only 35 participants (3% of the total smokers) had a confirmed diagnosis through spirometry, as shown in Fig. 1b and supplementary Table S3.

**Table 2:** Pulmonary function tests results among smokers

PFTs	Mean ± SD	Min – Max
FEV1%	83.4% ± 22%	7 − 200%
FVC %	86.6% ± 35%	9 − 206%
FEV1 / FVC %	82.6% ± 15%	20 − 120%

Gender, age, smoking years, and cigarette consumption per day were the statistically significant variables that affect lung function among smokers *p* = 0.009, 0.017, 0.015, 0.002, respectively, Table 3. In addition, there was a significant negative correlation between age, smoking years, and cigarette consumption per day variables and lung function FEV1/FVC ratio (*r* = -0.113; -0.270; -0.255, *p* < 0.0001) respectively. Significant predictors for developing confirmed COPD were male gender, smoking years 20–30, 10–20, > 20–30, > 30 cigarettes intake daily [OR 8.32, 1.58, 1.67, 2.52, 2.39] respectively. After applying multivariate logistic regression to adjust the OR, the significant predictors were identified as gender and daily cigarette consumption, while age showed no significance in either univariate or multivariate regression analyses, Table 4.

**Table 3:** Distribution of symptoms and factors affecting FEV1/FVC ratio among smokers

Variable, Frequency (%)	FEV1 / FVC %		Total (*n* = 1047)	*P*-value
	< 70% (*n* = 182)	≥ 70% (*n* = 865)		
Gender				
Male	181 (18%)	827 (82%)	1008	0.009*
Female	1 (3%)	38 (97%)	39	
Nationality				
Egyptian	180 (17%)	852 (83%)	1032	0.9
Non-Egyptian	2 (13%)	13 (87%)	15	
Age				
Less than 20 years	2 (50%)	2 (50%)	4	0.017*
20–29 years	26 (18%)	120 (82%)	146	
30–39 years	79 (16%)	423 (84%)	502	
40–49 years	41 (15%)	226 (85%)	267	
50–59 years	28 (25%)	85 (75%)	113	
≥ 60 years	6 (40%)	9 (60%)	15	
Smoking years				
Less than 20 years	117 (15%)	645 (85%)	762	0.015*
20–30 years	53 (22%)	185 (78%)	238	
More than 30 years	12 (25%)	35 (75%)	47	
Number of Cigarettes/ days				
Less than 10 cigarettes/day	28 (11%)	227 (89%)	255	0.002*
10–20 cigarettes/ day	84 (17%)	410 (83%)	494	
20–30 cigarettes/day	52 (24%)	167 (76%)	219	
More than 30 cigarettes/day	18 (23%)	61 (77%)	79	
Dyspnea on exertion				
Yes	94 (19%)	403 (81%)	497	0.221
No	88 (16%)	462 (84%)	550	
Sputum				
Yes	66 (17%)	333 (83%)	399	0.615
No	116 (18%)	532 (82%)	648	
Cough without cold				
Yes	62 (22%)	219 (78%)	281	0.17
No	120 (16%)	646 (84%)	766	
Your doctor ordered a PFT before				
Yes	10 (22%)	35 (78%)	45	0.419
No	172 (17%)	830 (83%)	1002	

PFTs: Pulmonary Function Tests

*Test of significance: Chi-square test or Fisher Exact test

N.B. These counts were mutually exclusive and the percentage was calculated by the total number within each row

**Table 4:** Univariate and multivariate logistic regression describing the significant predictors affecting obstructive lung disorders (FEV1 < 70%)

Predictor	Unadjusted OR [95%CI]	*P*	Adjusted OR [95% CI]	*P*
**Intercept**	------------	--------	0.02	< 0.001
**Gender (Male)**	8.32 [ 1.13; 60;97]	0.037	6,61 [1.89; 48.6]	0.046
**Smoking years**				
< 20 years 20–30 years > 30 years	Ref. 1.58 [ 1.09; 2.27] 1.89 [ 0.95; 3.75]	NA 0.014 0.068	Ref. 1.31 [0.90; 1.92] 1.49 [0.72; 3.01]	NA 0.155 0.283
**Cigarette daily intake number.**				
< 10 / day 10–20 /day > 20–30/ day > 30 / day	Ref. 1.67 [ 1.05; 2.62] 2.52 [ 1.53; 4.16] 2.39 [ 1.24; 4.61]	NA 0.031 < 0.001 0.009	Ref. 1.48 [0.93; 2.37] 2.15 [1.28; 3.61] 1.93 [1.01; 3.87]	NA 0.096 0.004 0.049

The statistical details for the model are as follows: Chi-square = 24.853, with a significance level of less than 0.001 (indicating high significance), degrees of freedom (df) = 6, and R-squared (R2) value of 4%. These variables were chosen to create the most reliable model, leading to the exclusion of age

NA: Not applicable

## Discussion

HLI was launched at COP27 and is still ongoing, with an analysis conducted for initial participants to underscore the importance of early detection, spotlighting adherence to PFTs and patient education, and convincing policymakers of the significance of this initiative. Our findings reveal that middle-aged males constitute the majority of smokers in Egypt, with nearly half experiencing dyspnea on exertion, which could significantly disrupt their daily activities during their most productive years. This aligns with the Woodruff et al. study, which observed that respiratory symptoms were present in 50% of current or former smokers with preserved pulmonary function. The average rate of respiratory exacerbations, as indicated by the COPD Assessment test (CAT score), among symptomatic smokers was notably higher compared to rates among asymptomatic current or former smokers and individuals who never smoked, suggesting a potential indication of underlying COPD [18]. Only a small fraction, 4.3%, of smokers underwent pulmonary function tests (PFTs), despite nearly two-thirds of them experiencing chronic respiratory symptoms. This discrepancy suggests a potential presence of undiagnosed COPD among smokers, even without confirmed spirometry results. These findings underscore the importance of further investigation for all smokers.

In our study, age did not emerge as a significant predictor for COPD development. However, the total number of smoking years and the number of cigarettes smoked per day, regardless of heavy smoking status, were significant factors. This finding is consistent with a recent study on a Spanish population, which included 8,819 individuals, 858 with COPD (FEV1/FVC < 70%) and 7,961 without. The study revealed that COPD risk increased with smoking duration, intensity, and lifetime tobacco consumption. Specifically, the risk increased for those with smoking durations of 50 years or more and smoking intensities of 39 cigarettes per day or more [19].

Moreover, the strong correlations between climate change and human behaviors, such as the prevalence of smoking and daily cigarette consumption, have become increasingly evident [20]. In our study, the percentage of adult participants who reported smoking 10–20 cigarettes per day at the 27th Conference of the Parties to the United Nations Framework Convention on Climate Change (UNFCCC) in Egypt was 47.2%. Smoking is considered a dual-edged risk because it may increase greenhouse gas emissions (CO_2_) and increase heat [21], as well as increase the impact of diseases (bronchial asthma, COPD). Environmental exposures impacted by climate change, such as air pollution, pollen, and other aeroallergens that are more retained in a warm climate, may further impede lung function [4]. Additionally, smoking is linked to lung cancer according to earlier research [22]. From the HLI administrative report, we identified that smoking has a high statistically significant risk for developing obstructive lung disease (FEV1/FVC ratio less than 70%), even after adjusting for age, gender, smoking years, and amounts by performing binary logistic regression [ OR = 15.4; *p* = 0.012, R2 = 51%].

The possible health threat posed by climate change has received little attention from American healthcare professionals. However, according to the American College of Physicians and Surgeons and 13 other national medical colleges from around the world, the biggest danger to human health in the twenty-first century is climate change, particularly for people with chronic lung diseases [23]. For the 12 million Americans who have COPD and other severe lung diseases, many aspects of climate change (increased heat, more intense weather events, and higher ozone levels) are more concerning. They also have a higher chance of dying as a consequence of climate change. Heat is associated with an increase in all-cause mortality, and heat waves particularly harm the elderly and those who have ongoing respiratory or cardiovascular conditions [24, 25]. For instance, during the 2006 heat wave in Portugal, for every 1 °C rise in mean temperature, all-cause mortality rose by 2.7% and COPD incidence rose by 5.4%. Even greater rises in COPD morbidity were seen in women and people over 75 [26]. A more significant impact modifier is air temperature because it can change the chemical composition of particulate matter. The effects of temperature on sulfate, organic carbon, and elemental carbon are all favorable [27]. Smoking prevalence during hot climates which are environmental stressors has a greater percentage of smokers [20]. Because smoking temporarily relieves the stress of the cold, smoking rates may rise in cold climates, contributing to climate warming and increasing risks. More research is required.

Pharmacological and psychological interventions have shown efficacy in aiding smoking cessation in Egypt. Furthermore, understanding psychological, personal, and social factors influencing smoking cessation can aid in tailoring interventions. However, despite various tobacco control efforts and policies, further measures such as standardized health education programs and stricter legislation are needed to prevent the rising smoking rates and meet global tobacco reduction targets [11]. HLI is a significant step in Egypt’s developing anti-smoking campaign that made a clear link between cigarette smoking and lung cancer and encouraged smoking cessation and stricter tobacco controls. Egypt is one of the top smoking nations in the world [28]. Today, reduced rates of tobacco-related morbidity and mortality have proven the benefits of those efforts. António Guterres, the secretary-general of the UN, urged everyone to continue “the battle for climate justice and climate ambition.” He concluded, “We can and must win this battle for our survival [29].

### Strengths of the study

This design provides valuable insights into the prevalence and predictors of COPD among smokers during the COP27 conference. The study included a diverse sample of participants attending the COP27 conference in Sharm El-Sheikh. This enhances the applicability of the study findings to a broader population with similar characteristics. The HLI employed an interview-based questionnaire and spirometry to collect data on various factors, including sociodemographic information, smoking history, respiratory symptoms, and pulmonary function tests. This comprehensive approach ensures a good assessment of COPD prevalence, predictors, and the adherence of physicians to ordering PFTs.

### Study limitations

The study primarily focused on smokers and their association with COPD. The data on non-smokers was not reported, and the study did not provide an in-depth analysis of COPD prevalence and predictors among non-smokers. Longitudinal or interventional studies would be needed to determine the causal effects of smoking on COPD and the impact of early detection through PFTs.

## Conclusion

The ongoing HLI, launched at COP27, emphasizes the vital importance of early detection, PFTs adherence, and patient education in combating smoking-related health risks, particularly among middle-aged males in Egypt. The correlation between smoking prevalence and climate change underscores the need for comprehensive strategies to mitigate both environmental and health impacts. The HLI serves as a pivotal initiative in Egypt’s anti-smoking efforts, demonstrating the tangible benefits of targeted interventions and stricter tobacco controls. Sustained efforts and collaboration are imperative to address the intertwined challenges of smoking and climate change, ensuring the safeguarding of public health and the achievement of climate justice.

### Electronic supplementary material

Below is the link to the electronic supplementary material.

Supplementary Material 1

## Data Availability

The datasets used and/or analyzed during the current study are available from the first or corresponding authors upon reasonable request.

## Abbreviations

°C: Celsius
CI: Confidence interval
CO2: Carbon Dioxide
COP27: 27th climate change conference
COPD: Chronic obstructive pulmonary disease
ED: Emergency Department
FER (FEV1/FVC): The forced expiratory volume in the first second to the forced vital capacity of the lungs
FER: Forced expiratory ratio
FEV1: Forced Expiratory Volume at the first second
FVC: Forced Vital Capacity
HLI: Healthy Lung Initiative
Max: Maximum
Min: Minimum
MoHP: Egyptian Ministry of Health and Population
OR: Odds ratio
P: P-value
PFTs: Pulmonary function tests
R2: Coefficient of determination
RTIs: Respiratory tract infections
SD: Standard deviation
UN: United Nations
UNFCCC: United Nations Framework Convention on Climate
US: United States
USA: United States of America
