# Supplementary material for: Revealing the significance of early detection of chronic obstructive pulmonary disease: insights from healthy lung initiative data at COP27 conference
Source: BMC Health Serv Res. 2024 Jul 31;24:872. doi: 10.1186/s12913-024-11107-8 (PMC11293106; doi:10.1186/s12913-024-11107-8)
Supplement: Supplementary file 1 — Supplementary Material 1 [file 12913_2024_11107_MOESM1_ESM.docx]

**S1:**  **Description of the demographics and characteristics of the initiative participants.**

| **Variable (N=1133)** | **Frequency** | **%** |  |
| --- | --- | --- | --- |
| **Gender “Male”** | **1087** | **95.9** |  |
| **Nationality “Egyptian”** | **1117** | **98.6** |  |
| **Age** |  |  |  |
| **Less than 20 years** | **4** | **4** |  |
| **20-29 years** | **147** | **13** |  |
| **30 – 39 years** | **557** | **49.2** |  |
| **40 -49 years** | **286** | **25.2** |  |
| **50 – 59 years** | **123** | **10.9** |  |
| **≥ 60 years** | **16** | **1.4** |  |
|  |  |  |  |
| **Smoking status “Yes”** | **1047** | **92.4** |  |
|  |  |  |  |
| **Smoking years** |  |  |  |
| **Nonsmoker** | **86** | **7.6** |  |
| **Less than 20 years** | **762** | **67.3** |  |
| **20 – 30 years** | **238** | **21** |  |
| **More than 30 years** | **47** | **4.1** |  |
| **Number of Cigarettes/ day** |  |  |  |
| **None** | **86** | **7.6** |  |
| **Less than 10 cigarettes/day** | **255** | **22.5** |  |
| **10 – 20 cigarettes/ day** | **494** | **43.6** |  |
| **20 – 30 cigarettes / day** | **219** | **19.3** |  |
| **More than 30 cigarettes / day** | **79** | **7** |  |
|  |  |  |  |
| **Dyspnea at effort “Yes”** | **509** | **44.9** |  |
| **Suffer from Sputum “Yes”** | **408** | **36** |  |
| **Cough without could “Yes”** | **290** | **25.6** |  |
| **Your doctor recommends PFT before “Yes”** | **48** | **4.2** |  |
| **FEV1/ FVC ratio “≥70%”** | **950** | **83.8** |  |
| **PFT: Pulmonary function test, FEV1: Forced expiratory volume at 1 second, FVC: Forced vital capacity.** | | |  |

**S2: Multivariate logistic regression describing the significant predictors for FEV1/FVC <70% for all Initiative participants (N= 1133).**

| **Predictor** | **Adjusted OR** | **CI** | **P-value** |
| --- | --- | --- | --- |
| **Intercept** | 0.0016 | 0.0004; 0.018 | <0.00001 |
| **Smoking status Yes** | 15.4 | 2.5; 295 | 0.012 |
| **Gender Male** | 8.7 | 1.6; 167 | 0.04 |
| **Age more than 60 years** | 3.7 | 1.1; 12.8 | 0.03 |
| **Cigarettes intake 20-30/ day** | 1.5 | 1.01; 2.7 | 0.049 |

**Table S3: Distribution of COPD symptoms among smokers categorized by spirometry confirmation status.**

| **Symptoms** | **Confirmed COPD (FEV1/FVC<70%)**  **n= 182** | | **Unconfirmed COPD (FEV1/FVC ≥70%)**  **n= 865** | | **Total**  **N= 1047** | |
| --- | --- | --- | --- | --- | --- | --- |
|  | **n** | **%** | **n** | **%** | **n** | **%** |
| **Dyspnea** | **41** | **23%** | **164** | **19%** | **205** | **19.6%** |
| **Chronic cough** | **21** | **12%** | **63** | **7%** | **84** | **8%** |
| **Significant Sputum** | **25** | **14%** | **177** | **20%** | **202** | **19.3%** |
| **Dyspnea & chronic cough** | **9** | **5%** | **36** | **4%** | **45** | **4.3%** |
| **Significant sputum & chronic cough** | **6** | **3%** | **18** | **2%** | **24** | **2.3%** |
| **Dyspnea & significant sputum** | **9** | **5%** | **68** | **8%** | **77** | **7.4%** |
| **Dyspnea & significant sputum & chronic cough** | **35** | **19%** | **147** | **17%** | **182** | **17.4%** |
| N.B. These counts were not mutually exclusive, and the percentage was calculated by the total smokerscolumn number. | | | | | | |
